# Association between Life's Crucial 9 and Cardiorenal syndrome: the mediating role of weight-adjusted-waist index

**DOI:** 10.3389/fnut.2025.1560224

**Published:** 2025-05-27

**Authors:** Huanjie Fu, Zhichao Liu, Hao Yu, Yisheng Zhao, Yongkang Gan, Jinhong Chen, Eryue Liu

**Affiliations:** ^1^Department of Cardiovascular, Second Affiliated Hospital of Tianjin University of Traditional Chinese Medicine, Tianjin, China; ^2^School of Rehabilitation Medicine, Shandong Second Medical University, Weifang, Shandong, China; ^3^Intensive Care Unit, Second Affiliated Hospital of Tianjin University of Traditional Chinese Medicine, Tianjin, China; ^4^Department of Vascular Surgery, Tianjin Academy of Traditional Chinese Medicine Affiliated Hospital, Tianjin, China; ^5^Beijing Key Laboratory of Drug Resistance Tuberculosis Research, Beijing Tuberculosis and Thoracic Tumor Research Institute, and Beijing Chest Hospital, Capital Medical University, Beijing, China

**Keywords:** Cardiorenal syndrome, Life's Crucial 9, weight-adjusted waist index, National Health and Nutrition Examination Survey, mediating role

## Abstract

**Background:**

Recent studies have indicated a link between cardiovascular wellbeing, obesity, and Cardiorenal syndrome (CRS). The impact of excessive body mass on the dynamics between heart health and CRS remains unclear. Life's Crucial 9 (LC9) serves as an innovative parameter for cardiac evaluation, whereas the Weight-Adjusted Waist Index (WWI) offers a nuanced metric for gauging obesity. This investigation explores the association between LC9 and CRS, and examines WWI's potential moderating influence.

**Methods:**

Data from the National Health and Nutrition Examination Survey (NHANES) was employed. Subgroup analyses were conducted, restricted cubic spline (RCS) modeling was utilized, and multivariate logistic regression was performed to assess the relationship between LC9 and CRS. Furthermore, we conducted a mediation analysis to investigate the influence of WWI on this relationship.

**Results:**

The cohort consisted of 25,379 participants, with 1,172 diagnosed with CRS. In the adjusted logistic regression model, an increase of ten points in LC9 correlated with a 25% reduction in CRS risk (OR = 0.75, 95% CI: 0.68, 0.82). Each incremental unit in WWI corresponded to a 63% increase in the risk of CRS (OR = 1.63, 95% CI: 1.46, 1.83). Tertile analysis of LC9 and WWI demonstrated consistent patterns, with significant *p-*values for trends < 0.001. RCS modeling confirmed a significant inverse linear correlation between LC9 and CRS (overall *p* < 0.001; non-linear *p* = 0.307) and a direct linear relationship between WWI and CRS (overall *p* < 0.001; non-linear *p* = 0.814). Mediation analysis revealed that WWI mediated 24.47% of the effect of LC9 on CRS (*p* < 0.001).

**Conclusion:**

The findings indicate a strong inverse relationship between LC9 and CRS, with WWI serving as a partial mediator in this interaction. The findings emphasize the intricate interactions between LC9 and CRS, illustrating the essential function of WWI as a mediator in future research endeavors.

## Introduction

Cardiorenal syndrome (CRS) encompasses a variety of conditions that affect the heart and kidneys, with impairment in one organ potentially leading to impairment in the other. This interaction is evident through various physiological mechanisms (1). Ongoing cardiac dysfunction can lead to renal impairment, while long-standing kidney disease may adversely affect cardiac function and increase the likelihood of cardiovascular issues. Roughly 15% of adults in the United States experience chronic kidney disease (CKD), while 2% are impacted by heart failure (HF), and 9% are affected by cardiovascular disease (CVD). As the population ages, the incidence of these diseases and associated comorbidities, like type 2 diabetes, is expected to rise (2). CRS is a significant component of the Cardiovascular-Kidney-Metabolic (CKM) syndrome, significantly impacting healthcare costs, disability, and mortality (3). Poor CKM health is associated with premature death and increased morbidity (4, 5). The pathophysiology of CRS is not yet completely understood, but several potential factors have been identified (6). However, a comprehensive understanding of these mechanisms remains elusive.

Current observations indicate a rising prevalence of excess body weight, with ~30% of the global population now categorized as overweight (7). In North America, approximately one-third of adults are classified as obese, with an accumulation of abdominal fat serving as a significant marker for metabolic disorders and CVD (8). While Body Mass Index (BMI) commonly assesses obesity, reflecting overall body fat rather than specifically abdominal fat, it is crucial to recognize that individuals with a normal BMI but elevated abdominal fat remain susceptible to negative health outcomes (9). To address this limitation, weight-adjusted waist index (WWI) was developed in 2018 (10) as a predictive measure for cardiometabolic diseases, CVD incidents, and all-cause mortality, offering superior accuracy (11, 12). Studies have demonstrated that WWI correlates with total, subcutaneous, and visceral fat, the latter of which has significant inflammatory properties, strengthening the link between total adiposity and CVD (13–15). Empirical evidence suggests that higher visceral fat levels increase the risk of CRS, while strategies to reduce abdominal fat may reduce the severity of CRS (16, 17).

Metabolic Syndrome (MetS), as defined by the World Health Organization (WHO), represents a complex pathological state characterized by the presence of abdominal obesity, insulin resistance, hypertension, and dyslipidemia (18, 19). A multitude of research efforts have consistently established a strong association between MetS and CVD, recognizing each component of MetS as a distinct predictor of cardiovascular risk (20). Moreover, studies have revealed a significant connection between CRS and MetS (21, 22). In 2010, the AHA established the Life's Simple 7 (LS7) framework to assess cardiovascular health (CVH) by examining particular behaviors and outcomes (23). The framework underwent an extension in 2022 to integrate mental health, resulting in Life's Essential 8 (LE8). In 2023, it was further refined into LC9, which encompasses sleep, smoking cessation, physical activity, diet, BMI, non-HDL cholesterol, blood glucose, blood pressure (BP), and mental health (24, 25). Previous studies suggest that a reduction in abdominal obesity may mitigate MetS (20, 26, 27). Given that both WWI and several factors of LC9 are amendable via lifestyle modifications (28), these components could pave new pathways for managing CRS. Recognizing that an increase in abdominal obesity accumulation may exacerbate the symptoms of chronic respiratory syndrome, and that weight management interventions alongside lifestyle changes encompass both metabolic and cardiovascular aspects, opens a pathway to enhance our comprehension of the dynamics involved in chronic respiratory syndrome.

This study suggests that WWI plays a mediating role in the relationship between LC9 and CRS, based on the findings presented. LC9, serving as a comprehensive marker of cardiovascular wellbeing, may provide protection against CRS by promoting health-oriented practices (such as a balanced diet and consistent physical activity) and enhancing clinical parameters. Nonetheless, obesity remains a crucial modifiable factor that may compromise the protective effect of LC9. Reflecting on fat distribution and its metabolic repercussions, WWI not only exhibits an independent association with CRS risk but may also act as a conduit linking LC9 to CRS. Through mediation analysis, this investigation endeavors to assess this hypothesis and unveil the mechanisms interlinking LC9, WWI, and CRS. These insights could be instrumental in devising preventive and therapeutic interventions for CRS. This study analyzes data collected from 2005 to 2018 through the NHANES to investigate the relationship between LC9 and CRS, while also exploring the mediating effect of WWI, which may contribute to improved strategies for the diagnosis and management of CRS.

## Methods

### Study participants

The NHANES, which is managed by the National Center for Health Statistics (NCHS), was used as a data source for this cross-sectional investigation. The National Center for Health Statistics' Research Ethics Review Board gave their stamp of approval to the NHANES protocols, and all participants gave their written consent. No further permission from the institutional review board was required for our secondary analysis because it followed the STROBE requirements for cross-sectional studies (29). Comprehensive details on NHANES methodologies and ethical guidelines are accessible via the Centers for Disease Control and Prevention (CDC) and NCHS websites. It is important to note that our study is based on the NHANES database, which is characterized by a large sample size and strong representativeness, making it a national-level survey. As a result, studies based on NHANES typically possess strong statistical power, enabling them to more effectively detect true associations between study variables. Therefore, in such studies, many literatures do not explicitly perform prospective sample size and statistical power calculations, which is common in NHANES-related research.

We analyzed nationally representative data from NHANES spanning 2005 to 2018. From the initial cohort of 70,190 participants spanning seven biennial cycles, we identified 39,038 individuals aged 20 years and older, excluding those who were pregnant. We further removed 13,639 respondents due to incomplete LC9 and WWI data and an additional 20 for insufficient CRS information, culminating in a study population of 25,379 participants (Supplementary Figure S1).

First, participants under the age of 20 were excluded because adolescents are in a stage of rapid physical development, which may result in significant differences in body composition and related health indicators. Including this group could increase heterogeneity in the analysis results. Second, for similar reasons, pregnant participants were also excluded. During pregnancy, women experience notable changes in physiological status and body composition, which could introduce confounding effects on the relationship between WWI, LC9, and CRS.

### CRS ascertainment

CRS encompasses a spectrum of cardiorenal disorders wherein acute or chronic dysfunction in one organ induces reciprocal dysfunction in the other (1). Criteria from a previous NHANES study defined CRS as the presence of both CVD and CKD at the same time (30). The presence of CVD was determined by self-reported diagnoses, which included conditions such as angina and CAD (31). The CKD-Epidemiology Collaboration (EPI) calculation was used to confirm CKD when the estimated glomerular filtration rate (eGFR) was < 60 mL/min per 1.73 m^2^ (32).

### Definition of WWI

The WWI, derived from NHANES data, measures central adiposity by combining waist circumference (WC) with weight using the formula WWI = WC (cm)/(weight (kg))^2^ (33). WWI served as the mediating variable in our analysis. Unlike BMI, which measures general adiposity, and the Waist-to-Height Ratio, which considers stature but not total weight, WWI integrates both WC and weight, offering a refined assessment of abdominal fat and its impact on CRS.

### Definition of LC9

Effective weight management, cholesterol moderation, glucose control, BP regulation, and mental health maintenance are the five physiological components that make up the LC9 index, which also includes four behavioral components: healthy eating, regular physical activity, non-smoking, and appropriate sleep. We used NHANES data to calculate each facet and gave it a score between 0 and 100. The total LC9 score is the mean of all nine metrics. As shown in Supplementary Table S2, the dietary quality was evaluated using the Healthy Eating Index-2015 (HEI-2015) (34). While trained NHANES personnel measured BMI, BP, glucose levels (GLuc), and cholesterol (LDL) in accordance with established protocols, data on sleep habits, cigarette usage, physical activity, and mental health were gathered from standardized questionnaire responses^1^.

### Co-variables

Age, gender, ethnicity, marital status, educational attainment, poverty-to-income ratio (PIR), and the occurrence of diabetes, hypertension, and hyperlipidemia were among the demographic and health factors that were used in our analysis. Supplementary Table S3 provides comprehensive descriptions of these variables.

### Statistical analysis

Statistical analyses utilized R software (version 4.3.1), applying sampling weights for national representativeness. The specific weighting variable used was “WTMEC2YR,” recalibrated for the 2005–2018 period as (1/7 × WTMEC2YR) (28). Data were expressed as mean ± standard deviation (SD) and analyzed with t-tests to compute *p-*values. The impact of LC9 and WWI on CRS was explored through three logistic regression models: (1) a crude model without covariate adjustment, (2) a model adjusted for age, sex, education level, marital status, PIR, and race, and (3) a model further adjusted for hypertension, diabetes, and hyperlipidemia. We employed a smoothing spline approach to investigate both linear and non-linear associations between LC9 and CRS. Subgroup analyses focused on the LC9-CRS relationship across various risk groups. We conducted a variance inflation factor (VIF) analysis using the vif() function from the “car” package in R for all covariates to assess the presence of multicollinearity. Generally, a VIF value below 10 indicates no severe multicollinearity. In our analysis, the VIF values for all covariates included in the regression model were significantly below this threshold. In this study, all VIF values were below 2, indicating that multicollinearity is not a concern in our research. Mediation analysis was conducted to determine the direct and indirect effects of WWI on the LC9-CRS linkage, calculating the mediated proportion by the formula: [indirect effect/(indirect effect + direct effect)] × 100%. To validate the robustness of our findings and assess the potential impact of missing data on our results, we conducted a sensitivity analysis. Specifically, we used the Multiple Imputation by Chained Equations (MICE) method to perform multiple imputations, generating five imputed datasets. We then repeated the primary analysis on the imputed datasets. The results were consistent with the original analysis, indicating that the handling of missing data did not substantially affect our conclusions (Supplementary Table S4). Mediation effects were estimated using the “mediation” package in R software (28, 35). Statistical significance was established at *p* < 0.05.

## Results

### Baseline characteristics

The analysis encompassed 25,379 participants aged 20 years or older, which is representative of ~279.7 million adults in the U.S. Among these, 3% were diagnosed with CRS, equivalent to an estimated 8.77 million individuals. Statistically significant disparities were noted in age, race, marital status, education level, income, and the prevalence of hypertension, diabetes, and hyperlipidemia between the CRS and non-CRS groups (*p* < 0.05). Those in the CRS cohort exhibited lower scores on the LC9 scale and higher WWI scores compared to their non-CRS counterparts (Table 1).

**Table 1:** Baseline characteristics of all participants were stratified by CRS, weighted.

**Characteristic**	**Overall, *N =* 279,654,111 (100%)**	**Non-CRS, *N =* 270,880,412 (97%)**	**CRS, *N =* 8,773,699 (3%)**	***p*-value**
No. of participants in the sample	25,379	24,207	1,172	–
**Age (%)**				**< 0.001**
*20–40*	101,234,596 (36%)	101,140,361 (37%)	94,235 (1.1%)	
*41–60*	108,714,719 (39%)	107,322,455 (40%)	1,392,264 (16%)	
*>60*	69,704,796 (25%)	62,417,596 (23%)	7,287,200 (83%)	
**Sex (%)**				0.059
*Female*	143,304,101 (51%)	139,113,097 (51%)	4,191,004 (48%)	
*Male*	136,350,010 (49%)	131,767,314 (49%)	4,582,695 (52%)	
**Race (%)**				**< 0.001**
*Non-Hispanic White*	197,151,539 (70%)	190,231,200 (70%)	6,920,339 (79%)	
*Non-Hispanic Black*	28,019,340 (10%)	27,041,045 (10.0%)	978,295 (11%)	
*Other*	32,839,900 (12%)	32,272,054 (12%)	567,846 (6.5%)	
*Mexican American*	21,643,331 (7.7%)	21,336,112 (7.9%)	307,220 (3.5%)	
**Married/live with partner (%)**				**< 0.001**
*No*	97,403,916 (35%)	93,517,277 (35%)	3,886,638 (44%)	
*Yes*	182,170,285 (65%)	177,283,224 (65%)	4,887,061 (56%)	
**Education level (%)**				**< 0.001**
*Below high school*	39,244,762 (14%)	37,026,106 (14%)	2,218,656 (25%)	
*High School or above*	240,289,721 (86%)	233,736,662 (86%)	6,553,059 (75%)	
**PIR (%)**				**< 0.001**
*Poor*	49,904,825 (19%)	47,909,929 (19%)	1,994,897 (24%)	
*Not Poor*	212,745,576 (81%)	206,503,579 (81%)	6,241,997 (76%)	
**Hypertension (%)**				**< 0.001**
*No*	174,171,352 (62%)	172,975,956 (64%)	1,195,396 (14%)	
*Yes*	105,482,759 (38%)	97,904,456 (36%)	7,578,303 (86%)	
**Diabetes (%)**				**< 0.001**
*No*	245,161,151 (88%)	240,490,944 (89%)	4,670,207 (53%)	
*Yes*	34,492,960 (12%)	30,389,467 (11%)	4,103,493 (47%)	
**Hyperlipidemia (%)**				**< 0.001**
*No*	80,992,857 (29%)	80,319,188 (30%)	673,668 (7.7%)	
*Yes*	198,661,254 (71%)	190,561,223 (70%)	8,100,031 (92%)	
LC9 [mean (SD)]	70.72 (13.54)	71.08 (13.38)	59.42 (13.53)	**< 0.001**
**LC9, Tertile (%)**				**< 0.001**
*T1*	96,873,654 (35%)	90,997,224 (34%)	5,876,430 (67%)	
*T2*	86,356,615 (31%)	84,306,086 (31%)	2,050,529 (23%)	
*T3*	96,423,841 (34%)	95,577,101 (35%)	846,740 (9.7%)	
WWI [mean (SD)]	10.96 (0.82)	10.93 (0.81)	11.73 (0.73)	**< 0.001**
**WWI, Tertile (%)**				**< 0.001**
*T1*	93,209,100 (33%)	92,644,615 (34%)	564,485 (6.4%)	
*T2*	93,245,978 (33%)	91,312,926 (34%)	1,933,052 (22%)	
*T3*	93,199,033 (33%)	86,922,871 (32%)	6,276,162 (72%)	

Mean (SD) for continuous variables: the p-value was calculated by the weighted Students t-test.

Percentages (weighted N, %) for categorical variables: the p-value was calculated by the weighted chi-square test.

LC9, Life's Crucial 9; WWI, Weight-adjusted waist index; PIR, poverty income ratio; CRS, Cardiorenal syndrome.

Bold values indicate *p* < 0.05.

### Association between LC9, WWI, and CRS

The results from three analytical models show that there is a continuous negative correlation between LC9 and the occurrence of CRS (*p* < 0.001), as shown in Table 2. The model 3 shows that the occurrence of CRS decreases by 25% for every 10-point increase in LC9 (Odds Ratio [OR]:0.75, 95% Confidence Interval [CI]:0.68, 0.82). A 47% decrease in CRS prevalence was observed in the highest LC9 tertile (T3), according to tertile analysis (OR: 0.53, 95% CI: 0.37, 0.76).

**Table 2:** Association between LC9, WWI, and CRS, NHANES 2005–2018.

**Characteristics**	**Model 1 [OR (95% CI)]**	***p*-value**	**Model 2 [OR (95% CI)]**	***p*-value**	**Model 3 [OR (95% CI)]**	***p*-value**
**LC9–CRS**						
Continuous (per 10 scores)	0.56(0.53,0.59)	< 0.001	0.61(0.56, 0.67)	< 0.001	0.75(0.68, 0.82)	< 0.001
**Tertile**						
T1	1 (ref.)		1 (ref.)		1 (ref.)	
T2	0.38(0.32,0.45)	< 0.001	0.46(0.38, 0.55)	< 0.001	0.62(0.52, 0.75)	< 0.001
T3	0.14(0.10,0.19)	< 0.001	0.25(0.18, 0.37)	< 0.001	0.53(0.37, 0.76)	< 0.001
*P for trend*	< 0.001		< 0.001		< 0.001	
**WWI–CRS**						
Continuous	3.26(3.01,3.53)	< 0.001	2.10(1.89, 2.33)	< 0.001	1.63(1.46, 1.83)	< 0.001
**Tertile**						
T1	1 (ref.)		1 (ref.)		1 (ref.)	
T2	3.47(2.51, 4.81)	< 0.001	1.67(1.18, 2.35)	0.004	1.19(0.84, 1.69)	0.320
T3	11.85(8.86,15.84)	< 0.001	3.28(2.37, 4.54)	< 0.001	1.86(1.32, 2.61)	< 0.001
*P for trend*	< 0.001		< 0.001		< 0.001	

Model 1: no covariates were adjusted.

Model 2: age, sex, education level, marital, and race were adjusted.

Model 3: age, sex, education level, marital, PIR, race, hypertension, diabetes, and hyperlipidemia were adjusted.

LC9, Life's Crucial 9; WWI, Weight-adjusted waist index; PIR, poverty income ratio; CRS, Cardiorenal syndrome; OR, odds ratio; CI, confidence interval.

**Figure 1:**
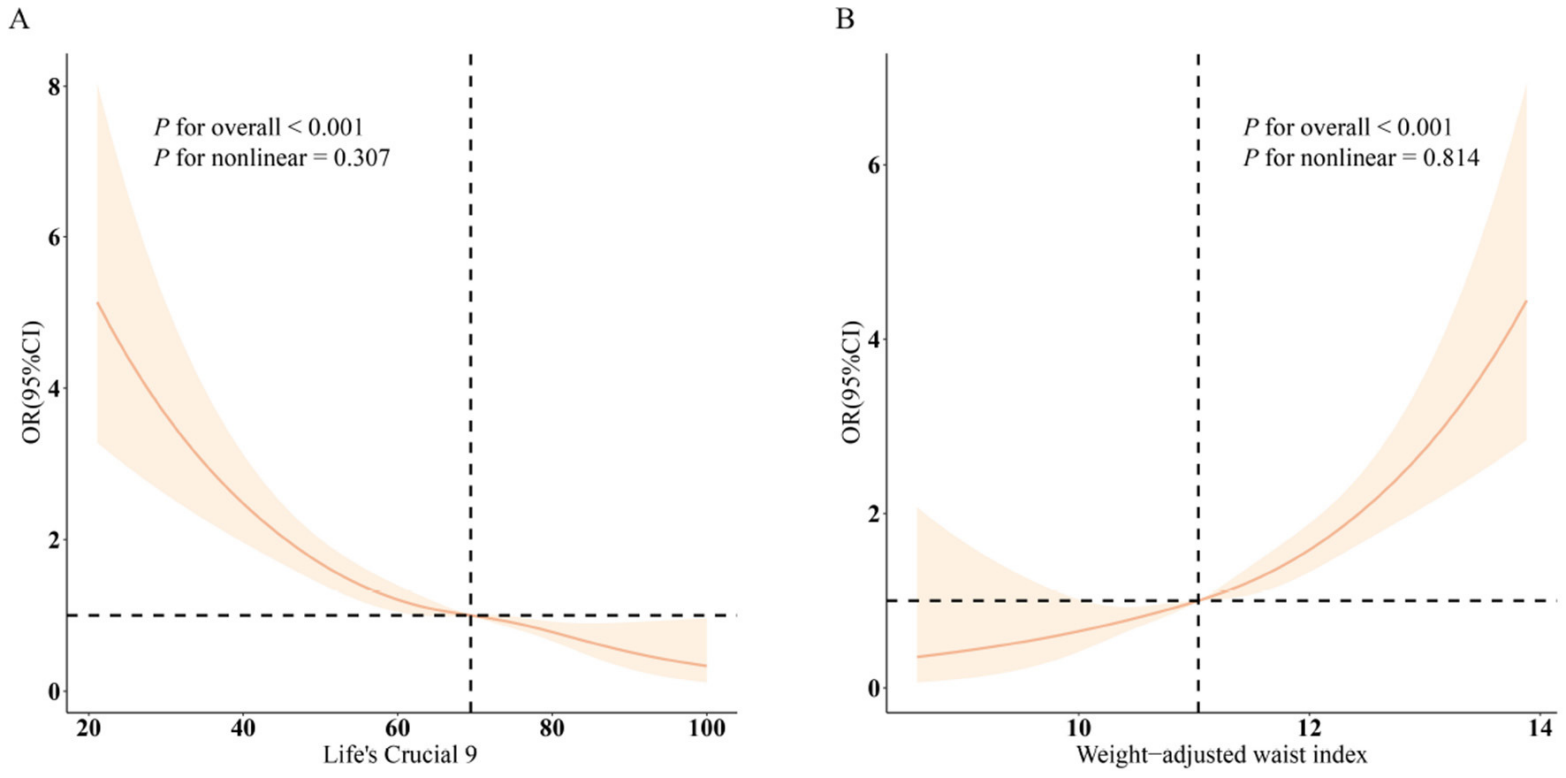
Dose-response relationships between LC9, WWI, and CRS. **(A)** LC9–CRS; **(B)** WWI–CRS. OR **(solid lines)** and 95% confidence levels **(shaded areas)** were adjusted for age, sex, education level, marital, PIR, race, hypertension, diabetes, and hyperlipidemia.

Conversely, an increase in WWI was associated with a rise in CRS prevalence across all models (*p* < 0.001). Specifically, a unit increment in WWI resulted in a 63% increase in CRS prevalence (OR = 1.63, CI: 1.46, 1.83). Higher WWI values consistently correlated with increased CRS prevalence (all *p* < 0.05). Restricted cubic spline (RCS) analyses (Figure 1A) reveal that LC9 maintains a linear negative association with CRS (overall *p* < 0.001; non-linear *p* = 0.307). In contrast, WWI exhibits a linear positive relationship with CRS (Figure 1B; overall *p* < 0.001; non-linear *p* = 0.814).

### Subgroup analyses

**Figure 2:**
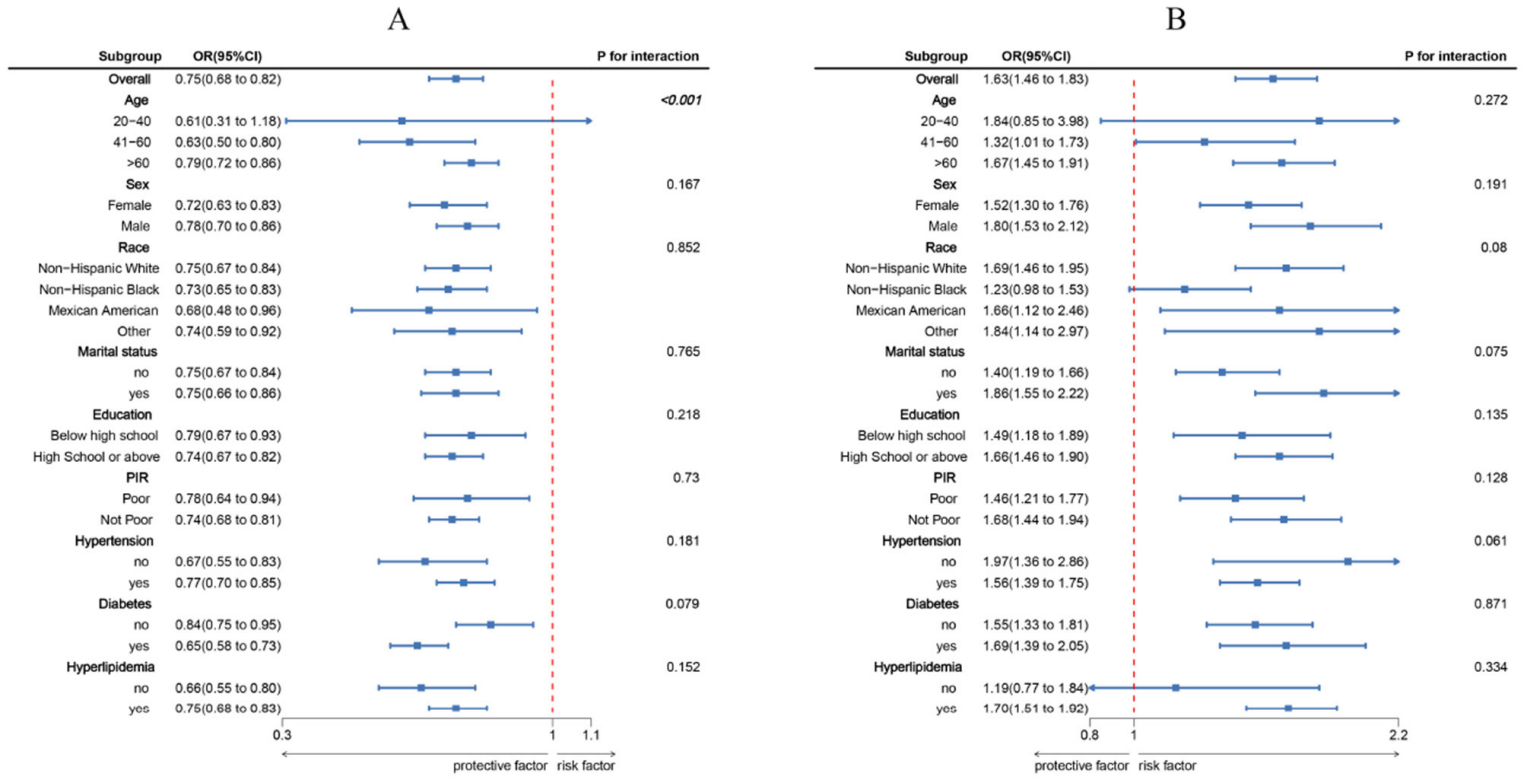
Subgroup analysis between LC9, WWI, and CRS. **(A)** LC9–CRS; **(B)** WWI–CRS. ORs were calculated per 10-unit increase in LC9, and each standard deviation increased in WWI. Analyses were adjusted for age, sex, education level, marital, PIR, race, hypertension, diabetes, and hyperlipidemia.

Figure 2 shows that there is a consistent inverse link between LC9 scores and CRS prevalence across subgroups characterized by age, gender, race, marital status, educational attainment, PIR, hypertension, diabetes, and hyperlipidemia. There was also a significant interaction (*p* < 0.05) between LC9 and age. The positive link between WWI and CRS prevalence was consistent across all subgroups that were studied.

### Mediation effect

**Figure 3:**
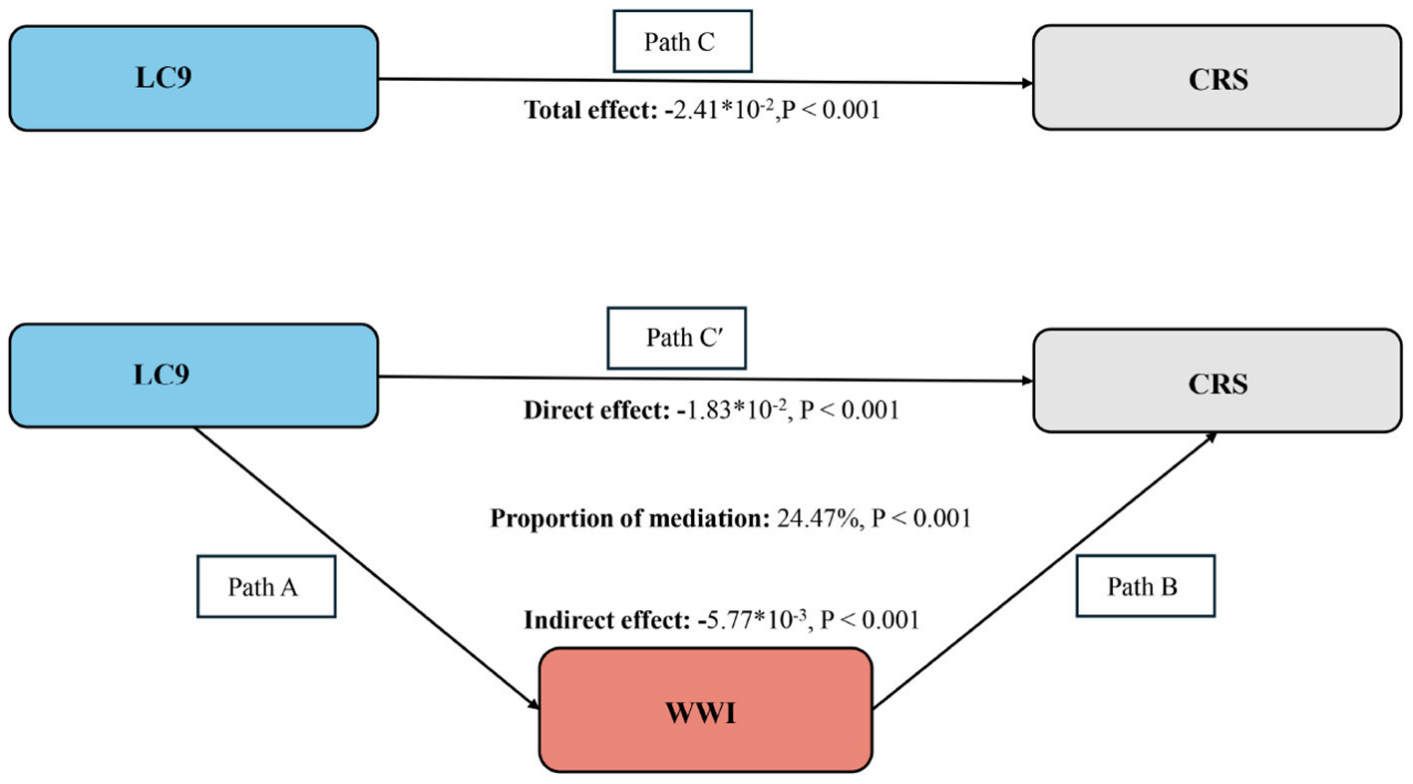
Schematic diagram of the mediation effect analysis. Path C indicates the total effect; path C′ indicates the direct effect. The indirect effect is estimated as the multiplication of paths A and B (path A*B). The mediated proportion is calculated as indirect effect / (indirect effect + direct effect) × 100%. LC9, Life's Crucial 9; WWI, Weight-adjusted waist index; CRS, Cardiorenal syndrome. Analyses were adjusted for age, sex, education level, marital, PIR, race, hypertension, diabetes, and hyperlipidemia.

Figure 3 depicts the mediation framework, identifying LC9 as the independent variable, CRS as the dependent variable, and WWI as the mediating factor. As illustrated in Table 3, a significant association between LC9 and WWI was confirmed following adjustments for covariates (β = −0.20, CI: −0.21, −0.19). After comprehensive adjustments, WWI clearly mediates the relationship between LC9 and CRS (indirect effect = −5.77 × 10^−3^, *p* < 0.001; direct effect = −1.83 × 10^−2^, *p* < 0.001), accounting for 24.47% of the effect (*p* < 0.001). Thus, WWI functions as a significant mediator in the LC9-CRS interaction.

**Table 3:** Multivariate linear regression of LC9 and WWI.

**Characteristics**	**β**	**95%CI**	***p*-value**
LC9–WWI	−0.20	(−0.21, −0.19)	< 0.001

Adjusted for age, sex, education level, marital, PIR, race, hypertension, diabetes, and hyperlipidemia.

## Discussion

Through the examination of data collected from 25,379 participants in the NHANES survey spanning from 2005 to 2018, our investigation revealed a negative correlation between LC9 scores and the occurrence of CRS, alongside a positive correlation between the WWI and CRS. Furthermore, mediation analyses revealed that WWI contributes to a portion of the association between LC9 and CRS. An interaction effect between LC9 scores and age was noted as well.

This study appears to be the first to investigate the relationship between LC9 and CRS, with WWI serving as a mediating factor. Prior studies have observed an inverse relationship between CVH, quantified by LE8, and the incidence of CRS (36, 37). WWI, recognized for its correlation with body fat levels and its implications for cardiometabolic health, indicates that tackling obesity could reduce the likelihood of metabolic conditions, such as chronic renal syndrome (11, 12, 38). Our findings align with these observations while further elaborating on them by integrating the influence of mental health within the interconnectedness of cardiovascular and renal health. The interplay between mental health factors, including anxiety, depression, and chronic stress, has been associated with an increased susceptibility to CVDs.

The LC9 elements may influence CRS through various biological pathways. Managing caloric intake, choosing high-fiber diets, and ensuring balanced nutrients and adequate hydration can reduce inflammation and improve metabolic regulation, thereby impacting CRS (39–41). Dietary modifications alter inflammatory markers and adiponectin expression through bioactive compounds that act as activators of nuclear hormone receptors and modulators of adiponectin secretion (42, 43). Increasing the intake of fiber and antioxidants may lessen systemic inflammation, potentially reducing CRS severity (44, 45). Diets featuring fruits, vegetables, and whole grains are thought to lower subclinical inflammation, which might alleviate CRS (46, 47). Enhanced fitness through physical activity may decrease cardiovascular events (48, 49) and moderate exercise can help modulate immune responses to reduce CRS risk (50). Physical activity also aids in weight management by limiting visceral adiposity, thereby counteracting CRS. Smoking promotes vascular stiffness, endothelial damage, fibrosis, and atherogenesis, each linked to CRS onset and worsening (51). Moreover, sleep quality impacts CRS because disorders in sleep or autonomic function can disrupt metabolism and homeostasis (52). The LC9 scoring approach underscores healthy sleep duration, potentially countering sympathetic overactivity and limiting CRS progression (53, 54). Compared to LE8, LC9 placed greater emphasis on the uniqueness and critical importance of mental health. Adequate rest can also alleviate mental health burdens, such as anxiety or depression, further improving symptoms (55). Mental health was associated with dysregulated energy-protein metabolism, which directly contributed to cardiorenal syndrome progression in longitudinal analyses (56).

Interaction analysis suggests a modifying role of age in the LC9–CRS relationship. Age-related comorbidities such as hypertension, diabetes, and dyslipidemia predispose individuals to cardiovascular and renal abnormalities, while aging itself is an independent risk factor (57, 58). Advancing age promotes endothelial dysfunction, oxidative stress, and inflammation, contributing to fibrotic changes, a recognized hallmark of cardiorenal disorders (59, 60). Potential pathophysiologic processes include extracellular matrix alterations, dysregulation of matrix metalloproteinases (e.g., MMP-9), and proinflammatory pathways that exacerbate organ aging (58). Additionally, the aging process may disturb the mTOR pathway, Klotho expression, and mitochondrial function, reinforcing CRS development (60, 61). Over time, arterial stiffening, vascular dysfunction, cognitive decline, and muscle loss can collectively raise morbidity and mortality among individuals with CRS (62).

The pathophysiology of CRS involves complex disease interactions (1). Chronic cardiac dysfunction results in diminished cardiac output, lowered blood flow, and increased venous pressure, subsequently affecting renal function. Compensatory mechanisms, including the activation of the renin–angiotensin–aldosterone system (RAAS) and the sympathetic nervous system, strive to sustain blood perfusion. Nevertheless, prolonged activation of the RAAS results in elevated aldosterone levels, which may contribute to harmful fibrosis and worsen the advancement of CVD and CKD (63). In instances of HF characterized by preserved ejection fraction, elements such as systemic inflammation and deficiencies in endothelial function, diastolic relaxation, and right ventricular performance contribute to the persistent nature of CRSs (64). Optimal interaction between cardiac and renal systems hinges on balanced neurohumoral feedback, water balance, and mitochondrial integrity (65, 66). Additionally, trimethylamine oxide (TMAO) has been linked to both cardiac and kidney abnormalities, which can affect outcomes in chronic HF and CKD, as well as overall population health (67).

WWI is a novel obesity indicator derived by adjusting waist circumference for body weight, aiming to better reflect the distribution of abdominal fat, particularly visceral fat. Numerous studies have demonstrated that WWI outperforms traditional obesity indicators in predicting the risk of chronic diseases. For instance, compared to BMI, WWI shows stronger associations with metabolic syndrome (68), asthma (69), cardiovascular diseases, and all-cause mortality (70). Therefore, we believe that WWI exhibits higher sensitivity in revealing obesity-related metabolic abnormalities. Given that the pathogenesis of cardio-renal syndrome is closely linked to visceral fat accumulation, systemic inflammation, and metabolic dysfunction, we selected WWI as a mediator variable to more precisely capture the mediating role of obesity between LC9 and CRS. WWI appears to mediate the LC9–CRS association partly because higher WWI suggests increased visceral fat and possibly lower muscle and bone mass. This pattern coincides with insulin resistance, dyslipidemia, and hyperglycemia (13, 71), each arising from RAAS activation, sympathetic overdrive, and inflammatory or oxidative pathways (9, 21). With aging, macrophages, adipocytes, and other cells accumulate in visceral adipose deposits, potentially responding to heightened metabolic demand, mitochondrial dysfunction, or DNA stress. These cells release pro-inflammatory molecules, chemokines, and proteases described as part of the senescence-associated secretory phenotype (SASP). This secretory profile aggravates adipose tissue inflammation, bringing in and activating immune cells (72). Meanwhile, better nutritional habits and more frequent physical activity—mainstays of LC9—can reduce visceral fat, improve BP, and stabilize lipid profiles (73, 74). Ultimately, this could indirectly reduce WWI and improve metabolic health, thereby lowering the risk of CRS.

Our study has numerous advantages: (1) It is the first to address the link between LC9 and CRS in an American population, suggesting that LC9 could become a powerful clinical indicator for CRS. (2) WWI is introduced as a novel measure of visceral adiposity, outperforming conventional anthropometrics in pinpointing at-risk patients at minimal cost. (3) We leveraged extensive data from NHANES (2005–2018), capturing a nationally representative sample. (4) Multiple modeling techniques and subgroup analyses were used to adjust for confounders, highlighting a persistent negative association between LC9 and CRS and a positive association between WWI and CRS. (5) Subgroup analysis suggests that age modifies the LC9–CRS interplay (*p* < 0.05), indicating further work is needed to confirm these observations.

Nonetheless, the ramifications of our results are limited by various constraints: (1) The cross-sectional design of the study restricts our capacity to draw causal inferences regarding the relationship between LC9 and the prevalence of CRS; a more extensive, prospective cohort study would more effectively clarify temporal associations. This study is cross-sectional in nature, making it unable to establish the temporal sequence among LC9, WWI, and CRS. Additionally, unmeasured or inadequately controlled confounding factors, such as genetic predisposition, long-term dietary patterns, or other metabolic abnormalities, may still interfere with the observed associations. Furthermore, it cannot be ruled out that the presence of CRS might conversely affect individuals' cardiovascular health behaviors or fat distribution, potentially leading to issues of reverse causation. (2) The NHANES utilizes a stratified sampling technique to accurately reflect the non-institutionalized population of the United States, yet it does not include individuals who are hospitalized or residing in long-term care facilities, thereby limiting the broader applicability of our findings. The absence of participation in surveys or the occurrence of incomplete assessments may lead to the introduction of selection bias. (3) CRS case ascertainment here depended on self-reported diagnoses within NHANES, which may be prone to recall error. (4) In using NHANES data, we accessed a substantial dataset but could not entirely rule out confounding or fully untangle relationships among the different LC9 components. Discrepancies in data quality necessitate rigorous statistical strategies to ensure reliability. Future work might investigate interactions among these variables or apply advanced analytic methods to mitigate confounding. Additionally, combining the CRS Symptom Scale (CRSSS) with other clinical instruments or measures might offer a more exhaustive view of CRS symptomatology and life quality. We also recommend refining diagnostic criteria to account for symptom frequency, severity, duration, and effect on wellbeing. (5) Prospective, longitudinal data and precise diagnostic benchmarks will be needed to strengthen the identification of CRS. (6) NHANES data exclude hospitalized patients and long-term care populations, which may lead to limited extrapolation of results. (7) We are fully aware of the limitations of WWI itself. First, its calculation is based on waist circumference and body weight, making it susceptible to measurement errors. Second, as a relatively novel indicator, WWI has not yet established uniform clinical reference values across all populations, and its applicability across different races and age groups requires further validation. Taken together, although some caveats remain, our study contributes crucial evidence regarding the interplay of LC9, WWI, and CRS. Addressing the noted limitations will improve the reliability, translation, and utility of subsequent research in this area.

## Conclusion

Our analyses reveal a robust inverse correlation between LC9 and CRS, with WWI acting as a partial mediator. This observation highlights a possible connection between CVH and CRS, stressing the importance of managing obesity in this context. Our findings offer novel perspectives on both preventing and managing CRS, suggesting that a multifaceted strategy to enhance CVH and reduce obesity might help diminish CRS prevalence. Looking ahead, prospective investigations will be vital for elucidating the detailed mechanisms behind these links. Moreover, future work could delve deeper into other risk factors, including mental health challenges, that may also shape this association.

## Data Availability

The datasets presented in this study can be found in online repositories. The names of the repository/repositories and accession number(s) can be found at: https://www.cdc.gov/nchs/nhanes/index.htm.

## Footnotes

^1^ https://www.cdc.gov/nchs/nhanes/index.htm
